# Dichotomous role of Shp2 for naïve and primed pluripotency maintenance in embryonic stem cells

**DOI:** 10.1186/s13287-022-02976-z

**Published:** 2022-07-18

**Authors:** Seong-Min Kim, Eun-Ji Kwon, Yun-Jeong Kim, Young-Hyun Go, Ji-Young Oh, Seokwoo Park, Jeong Tae Do, Keun-Tae Kim, Hyuk-Jin Cha

**Affiliations:** 1 College of Pharmacy, Seoul National University, 1 Gwanak-ro Gwanak-gu, Seoul, 08826 Republic of Korea; 2 Research Institute of Pharmaceutical Sciences, Seoul National University, Seoul, Republic of Korea; 3 Department of Biomedical Sciences, Seoul National University College of Medicine, Seoul, South Korea; 4 Department of Stem Cell and Regenerative Biology, College of Animal Bioscience and Technology, Konkuk University, Seoul, Republic of Korea

**Keywords:** Ptpn11, Shp2, Tyrosine phosphatase, Naïve pluripotency, Self-renewal, Mek1, 2i/LIF, Embryonic stem cells, Primed pluripotency, Erk1/2

## Abstract

**Background:**

The requirement of the Mek1 inhibitor (iMek1) during naïve pluripotency maintenance results from the activation of the Mek1-Erk1/2 (Mek/Erk) signaling pathway upon leukemia inhibitory factor (LIF) stimulation.

**Methods:**

Through a meta-analysis of previous genome-wide screening for negative regulators of naïve pluripotency, *Ptpn11* (encoding the Shp2 protein, which serves both as a tyrosine phosphatase and putative adapter), was predicted as one of the key factors for the negative modulation of naïve pluripotency through LIF-dependent Jak/Stat3 signaling. Using an isogenic pair of naïve and primed mouse embryonic stem cells (mESCs), we demonstrated the differential role of Shp2 in naïve and primed pluripotency.

**Results:**

Loss of Shp2 increased naïve pluripotency by promoting Jak/Stat3 signaling and disturbed in vivo differentiation potential. In sharp contrast, Shp2 depletion significantly impeded the self-renewal of ESCs under primed culture conditions, which was concurrent with a reduction in Mek/Erk signaling. Similarly, upon treatment with an allosteric Shp2 inhibitor (iShp2), the cells sustained Stat3 phosphorylation and decoupled Mek/Erk signaling, thus iShp2 can replace the use of iMek1 for maintenance of naïve ESCs.

**Conclusions:**

Taken together, our findings highlight the differential roles of Shp2 in naïve and primed pluripotency and propose the usage of iShp2 instead of iMek1 for the efficient maintenance and establishment of naïve pluripotency.

**Supplementary Information:**

The online version contains supplementary material available at 10.1186/s13287-022-02976-z.

## Background

Although mouse embryonic stem cells (mESCs) have been widely used to characterize the early embryonic development of mammals, these cells exhibit clear differences from human ESCs at the cellular (e.g., colony morphology and surface markers) [1] and molecular levels, such as different epigenetic states, X-chromosome inactivation, chimera formation, major signaling pathways [2, 3], and glucose metabolism [4], among others [5]. Based on recent advances in our understanding of pluripotency, two discrete pluripotent states, naïve (or ground) and primed, have been recognized. In more detail, “naïve” and “primed” cells represent the peri-implanted blastocyst, at E4.5 and post-implanted embryo (from E5.5 onwards) [6]. Since naïve ESCs are established from human ESCs [7], dissimilarities of human to mouse ESCs have been explained to be unique characteristics of primed pluripotency [8].

A constant supply of leukemia inhibitory factor (LIF) along with two chemical inhibitors for Mek1 (iMek1) and Gsk3β (iGsk3β) (hereinafter referred to as LIF + 2i) is indispensable [9] not only for maintenance but also for the establishment of naïve ESCs, thus mimicking the characteristics of naïve pluripotency of pre-implantation embryos in vitro. LIF-mediated propagation and maintenance of naïve ESCs depend on the Jak/Stat3 pathway [10], which is also conserved in embryo development and implantation [11]. Two inhibitors are required for maintaining naïve pluripotency, which is likely attributed to the innate mechanism of Gsk3α/β inhibition in the inner cell mass (ICM) of blastocysts [12] coupled with a lower basal activity of Erk1/2 (hereinafter Erk) in early blastocysts [13], the latter of which is possibly due to the expression of Erk-specific dual-specificity phosphatases (DUSPs) [14, 15].

Src homology region 2 domain-containing phosphatase 2 (Shp2), which is encoded by protein tyrosine phosphatase non-receptor type 11 (*Ptpn11*), transmits the receptor signaling to Erk [16] and negatively modulates Jak/Stat3 signaling [17]. Phosphorylation of Shp2 upon cytokine stimulation by interleukin-6 (IL-6) or LIF, leads to intramolecular conformational changes to relieve autoinhibition of tyrosine phosphatase activity [18] and dephosphorylates the Jak/Stat3 pathway [18, 19]. In parallel, the recruitment of phosphorylated Shp2 to gp130 forms a protein complex with Gab1 to transduce signals from the receptor to Ras/Raf/Mek/Erk [20]. Considering the positive role of Shp2 in Ras-dependent Erk activation (Ras-to-Erk), as well as in oncogenic Ras-to-Erk [21, 22] and target therapy resistance [23], small molecules to inhibit the bivalent roles of Shp2 have been developed as novel anti-cancer therapeutic agents [24].

As negative roles of Shp2 in self-renewal of mESCs through Erk [25], Shp2 deficiencies promote self-renewal capacity and suppress mESC differentiation through increased sensitivity to LIF on Jak/Stat3 and attenuation of Ras-to-Erk [17]. Similarly, failure to recruit Shp2 [26] and Shp1 [2] to the site of Stat3 activation sensitizes mESCs to LIF in the Jak/Stat3 signaling pathway, thus favoring self-renewal. In contrast, the molecular mechanisms that control the pluripotency and differentiation of hESCs via SHP2 depletion are different from those of mESCs [27], which suggests the dichotomous role of Shp2 in naïve and primed ESCs.

Here, we demonstrated that transient activation of Shp2 by LIF negatively modulated Jak/Stat3 signaling and transmitted the signal to Ras-to-Erk, which consequently resulted in an attenuation of naïve pluripotency. Accordingly, depletion of Shp2 or iShp2 (a chemical inhibitor of Shp2) treatment favors naïve pluripotency by sensitizing the Jak/Stat3 signaling pathway to LIF stimulation and decoupling Ras-to-Erk signaling, whereas primed ESCs were intolerant to Shp2 perturbation. The dichotomous effect of Shp2 inhibition thus favored the enrichment of naïve ESCs. Additionally, iShp2 could efficiently substitute iMek1 for the maintenance and establishment of naïve pluripotency.

## Results

### Shp2 activation by LIF negatively affects naïve pluripotency

**Fig. 1:**
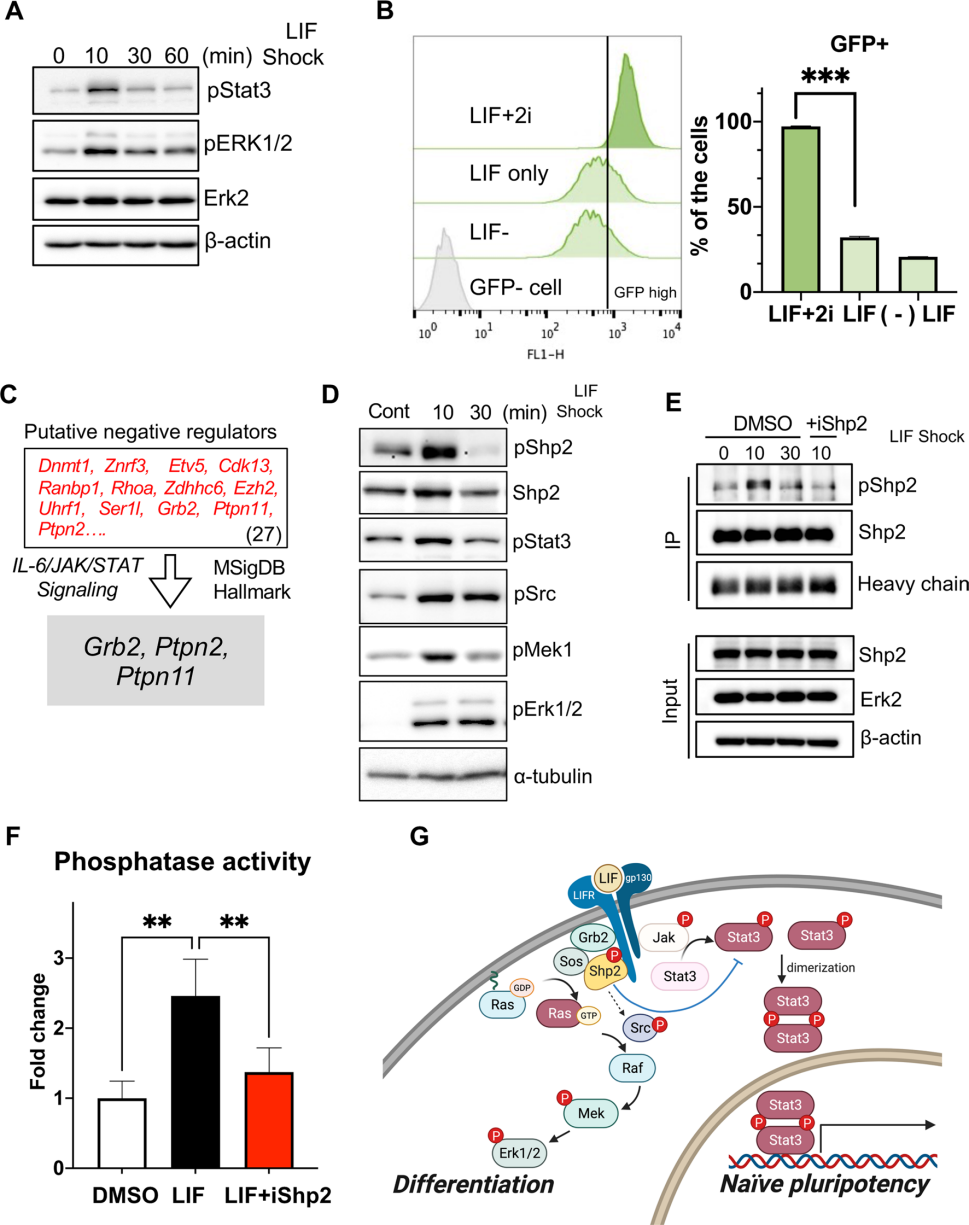
Shp2 activation by LIF negatively affects naïve pluripotency. **A** Immunoblotting at indicative time after LIF stimulation (1000 U/ml), Erk2 and β-actin were used as loading controls. **B** Flow cytometry of GFP after 60 h stimulated by LIF + 2i, LIF only, LIF deprivation (LIF-) media respectively (left panel), Graphical presentation of GFP positive population (right panel) (***p < 0.001, *n* = 3). **C** List of 27 genes that were predicted as putative negative regulators of naïve pluripotency (top), gene list involved in ‘IL-6/JAK/STAT3 Signaling Pathway’ (bottom). **D** Immunoblotting for indicative proteins at 10 and 30 min after LIF stimulation (1000 U/ml), α-tubulin was used as a loading control. LIF starvation for 1 h was performed before the LIF stimulation. **E** Immunoblotting for indicative proteins at 10 and 30 min after LIF stimulation (1000 U/ml), β-actin was used as loading control. LIF starvation for 1 h was performed before the LIF stimulation. DMSO (Mock) or iShp2 (5 μm) was treated while LIF starvation. **F** Graphical presentation of phosphatase activity of OG2 mESCs at 10 min after LIF stimulation with or without iShp2 (5 μm) for 1 h (**p < 0.01, *n* = 4). **G** Graphical illustration of LIF mediated signaling of naïve ESCs

Upon LIF stimulation following LIF deprivation, we observed that Erk activation occurred along with Stat3 phosphorylation (Fig. 1A). To determine the occurrence of naïve pluripotency, we took advantage of OG2 mESCs, which express naïve specific green fluorescent protein (GFP) under the control of the Oct4 promoter due to the lack of a proximal enhancer [28, 29]. Consistent with previous reports [6], a lack of 2i resulted in the loss of the dome shape that characterizes naïve specific colony morphology (Additional file 1: Fig. S1A, Additional file 2: Movie S1A, Additional file 3: Movie S1B, Additional file 4: Movie S1C) and significantly attenuated GFP signals even under LIF stimulation (Fig. 1B). Due to innate negative regulator(s) toward Stat3 [30], Stat3 phosphorylation after LIF stimulation (Fig. 1A) and its transcriptional activity (Additional file 1: Fig. S1B) were attenuated along with Erk activation through a series of protein interactions. Therefore, the putative negative feedback mechanism(s) and/or Erk activation upon LIF stimulation, would be responsible for the constant supply of LIF + 2i for maintaining naïve pluripotency. In a previous study, genome-wide CRISPR screening identified a set of negative and positive regulators of naïve pluripotency [31]. A total of 155 genes (Additional file 1: Fig. S1C and Additional file 9: Table S1), whose perturbation significantly affected naïve pluripotency [31], were examined via gene ontology (GO) and KEGG pathway analysis. As expected, based on the significance of LIF-mediated signaling on naïve pluripotency described in other studies, these 155 genes were highly enriched in several associated pathways including ‘Interleukin-6 signaling,’ ‘Jak/Stat3 signaling,’ and ‘PluriNetWork’ (Additional file 1: Fig. S1D). Particularly, a few genes among a set of 27 positive regulators (shown in red) and 128 negative regulators (shown in blue) were associated with the ‘Naïve pluripotency signaling’ (Additional file 1: Fig. S1E), ‘JAK/STAT signaling’ (Additional file 1: Fig. S1F), and ‘Ras signaling’ (Additional file 1: Fig. S1G) pathways. Among 27 putative negative regulators, three genes including *Grb2, Ptpn2,* and *Ptpn11* belonged to the “IL-6/JAK/STAT signaling” pathway according to MSigDB Hallmark 2020 (https://maayanlab.cloud/Enrichr/) [32] (Fig. 1C). We next focused on *Ptpn11*, which encodes the Shp2 protein that dephosphorylates Stat3, thus acting as an important negative regulator of IL-6 stimulation [33], and transduces signals toward Ras-to-Erk possibly through Grb2 [34]. Considering the close similarity between LIF and IL-6 [35], we presumed that the activation of Shp2 by LIF would act as a negative regulator on naïve pluripotency by Stat3 dephosphorylation and Erk1/2 activation. As predicted, active phosphorylation of Shp2 (Y542) occurred promptly after LIF stimulation in parallel with signal transduction to Ras-to-Erk (Fig. 1D), thus relieving its auto-inhibitory regulation on phosphatase activity [18]. In parallel with Shp2 active phosphorylation (Fig. 1D), the phosphatase activity of Shp2 [about 45% inhibited by Shp2 inhibitor (iShp2) treatment], was also clearly induced by LIF stimulation (about 60% increased) (Fig. 1F). These data suggest that Shp2 activation by LIF simultaneously controls both Jak/Stat3 and Ras-to-Erk signaling to affect naïve pluripotency (Fig. 1G).

### Role of Shp2 in naïve pluripotency

**Fig. 2:**
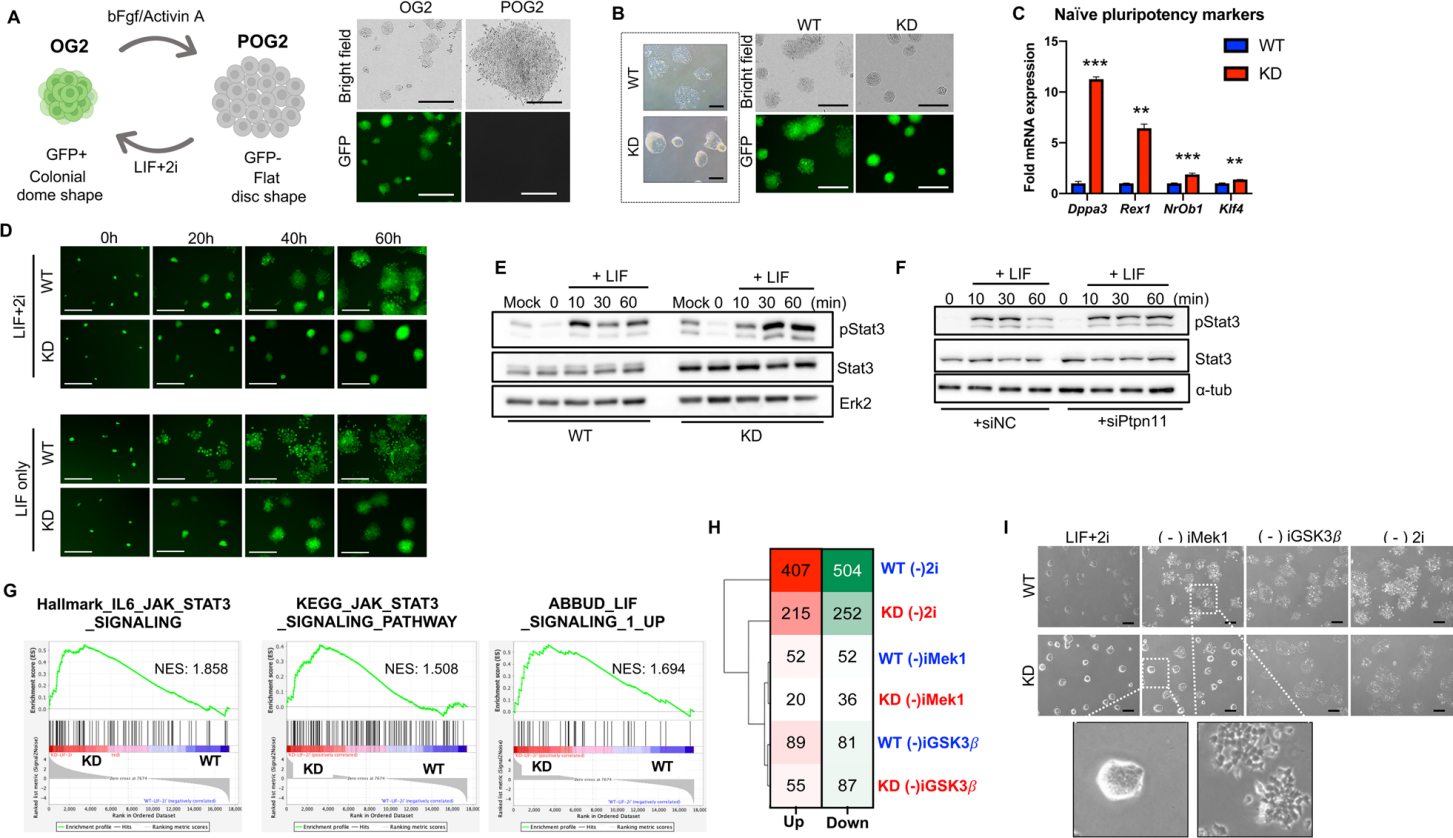
Role of Shp2 in naïve pluripotency. **A** Graphical illustration of isogenic pair of naïve (OG2) and primed (POG2) ESCs (left), brightfield and fluorescent microscopic images of OG2 and POG2 ESCs (scale bars, 500 μm) (right panel). **B** Representative light microscopic images of WT and KD ESCs (scale bars, 200 μm) (left panel) and fluorescent microscopic images of WT and KD ESCs (scale bars, 500 μm) (right panel). **C** Fold mRNA expression of typical naïve pluripotency marker genes in WT and KD ESCs (**p < 0.01 and ***p < 0.001, *n* = 3). **D** Fluorescent microscopic images of WT and KD ESCs at indicated time under LIF + 2i and LIF only culture condition (scale bars, 500 μm). **E** Immunoblotting for pStat3, Stat3 and Erk2 in WT and KD ESCs at indicated time after LIF stimulation (1000 U/ml), Erk2 was used as a loading control. LIF starvation for 1 h was performed before the LIF stimulation. **F** Immunoblotting of OG2 mESCs with control (siNC) or Ptpn11 siRNA (siPtpn11) at indicated time after LIF stimulation (1000 U/ml), α-tubulin was used as a loading control. LIF starvation for 1 h was performed before the LIF stimulation. **G** The normalized enrichment score by gene set enrichment analysis (GSEA) of WT and KD ESCs for Hallmark_IL6_JAK_STAT3 signaling (left panel), KEGG_JAK_STAT3 signaling (middle panel), and ABBUD_LIF signaling up (right panel). **H** DEGs of WT LIF + 2i vs WT (-)2i were assigned as ‘gene sets for 2i’. The number of Up-DEGs and Down-DEGs within the ‘gene sets for 2i’ group are presented. **I** Light microscopic images of WT and KD 48 h after indicated culture condition (scale bars, 200 µm)

**Fig. 3:**
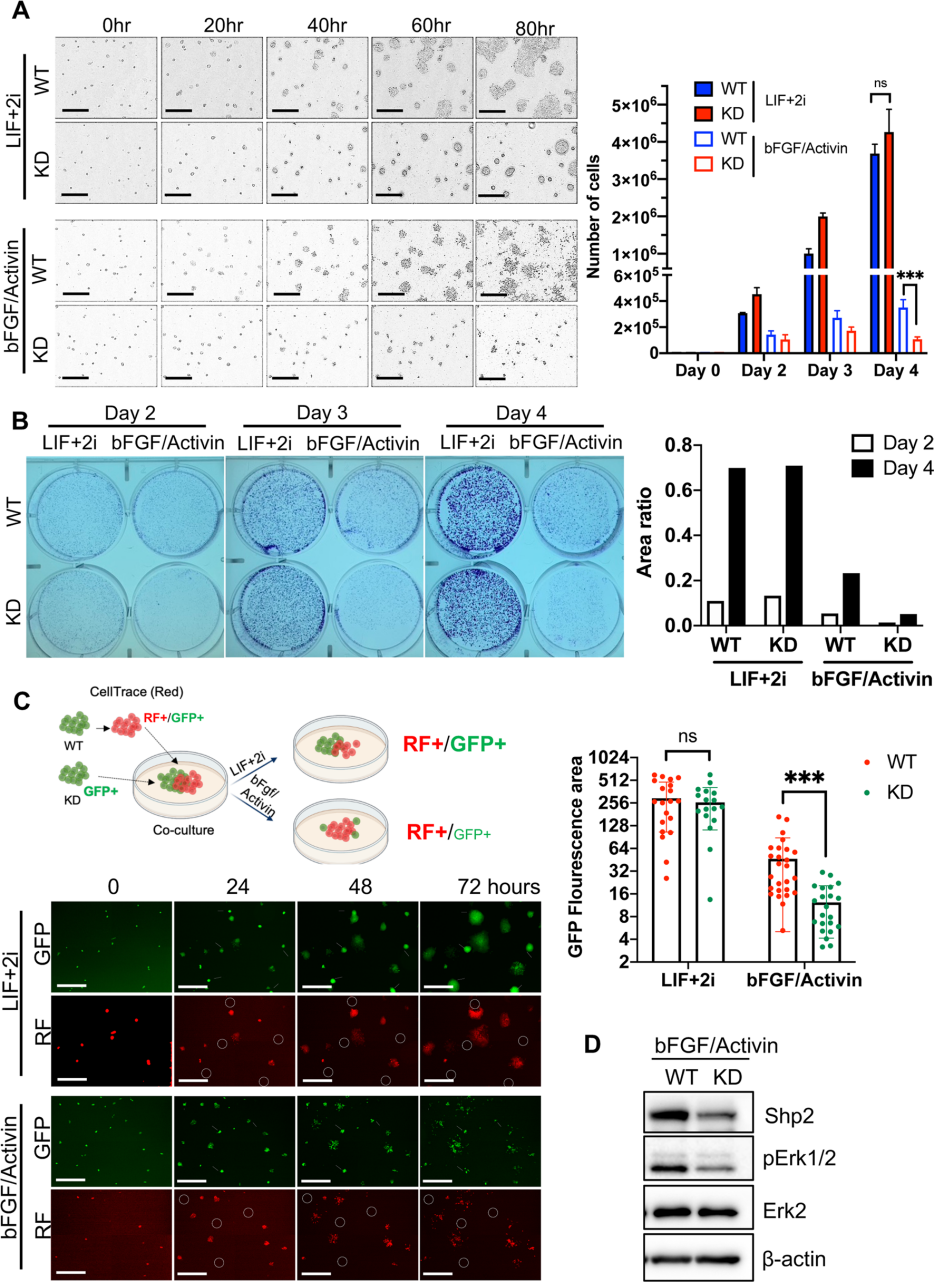
Role of Shp2 in primed pluripotency. **A** Light microscopic images of WT and KD ESCs at indicated times under LIF + 2i or bFGF/Activin culture condition (scale bars, 500 μm) (left), Graphical presentation of cell number at indicated time after seeding of 1 × 10^4^ ESCs (right). **B** Images of clonogenic assay of WT and KD ESCs at indicated time under LIF + 2i or bFGF/Activin culture condition (left), Graphical presentation of area ratio (A.U.) of clones at day 2 and day 4 determined by ImageJ (right). **C** Graphical illustration of competition assay with WT, labeled with red fluorescence [RF/GFP], and KD [GFP only] mESCs (top), Fluorescence microscopic images of WT or KD ESCs at indicated time after each culture condition, circles indicate KD ESCs [GFP only] (scale bars, 500 μm) (left), Relative fluorescence intensity of WT (red) and KD (green) ESCs from GFP images from 72 h was presented as a graph (right) (***p < 0.001, *n* = 26). **D** Immunoblotting analysis of WT and KD ESCs grown under bFGF/Activin for Shp2, pStat3, pErk1/2, Erk2 and β-actin. β-actin was used as a loading control

Although the roles of Shp2 were well-characterized in self-renewal of mESCs with knockdown (KD) or knockout (KO) models [17], we noticed that the effect of Shp2 in human ESCs (hESCs) was less evident than that of mESCs [27]. Given that hESCs share the molecular and cellular characteristics of primed ESCs [8], we hypothesized that the effect of Shp2 in naïve pluripotency would differ from that of primed pluripotency. To evaluate this hypothesis, we utilized the isogenic model of pair of primed ESCs (P-OG2) and naïve ESCs (OG2). As previously described [29], naïve ESCs with a ‘colonial dome shape’ exhibit GFP signals unlike primed ESCs, which exhibit a ‘flat disc shape’ (Fig. 2A), expressing typical marker genes of the naïve and primed state (Additional file 1:Fig. S2A and B). Stable knockdown of *Ptpn11* was performed using a pair of naïve and primed ESCs. As expected, based on previous studies performed in mESCs, naïve ESCs with clear *Ptpn11* knockdown (hereinafter referred to as Shp2 KD or KD naïve ESCs) (Additional file 1:Fig. S2C) exhibited a clear ‘colonial dome shape’ with an increased GFP signal (Fig. 2B). Consistently, naïve cell-specific marker genes were also significantly enhanced in KD Naïve ESCs (Fig. 2C), whereas core pluripotency genes only exhibited marginal changes (Additional file 1:Fig. S2D). Interestingly, KD Naïve ESCs maintained a stable GFP signal even under LIF treatment without 2i supplement (LIF only), which significantly affected the aforementioned ‘colonial dome shape’ morphology (Additional file 1:Fig. S2E) as well as the GFP signal of wild-type (WT) cells (Fig. 3D). Based on the drastic increase in active phosphorylation and phosphatase activity (Fig. 1D and E) of Shp2 by LIF, the prolonged naïve characteristics of KD naïve ESCs under the LIF-only condition resulted from sustained Stat3 phosphorylation via stable (Fig. 2E) and transient (Fig. 2F) Shp2 depletion. These results were also supported by the gene set enrichment analysis (GSEA) [36] of FPKM values from the WT and KD transcriptomes. Consistently, the gene set for ‘Hallmark IL6 JAK STAT3 signaling’, ‘KEGG JAK STAT3 signaling pathway,’ and ‘LIF signaling 1 UP’ were significantly enriched in the KD cells compared to their WT counterparts (Fig. 2G). However, we still could not fully account for the marginal effect of 2i withdrawal on GFP signals (Fig. 2D and Additional file 5: Movie S2A, Additional file 6: Movie S2B. Additional file 7: Movie S2C. Additional file 8: Movie S2D), as well as the morphological changes of KD, illustrated in Additional file 1:Fig. S2E. Therefore, the transcriptomes of WT and KD naïve ESCs after depletion of iMek1 or iGsk3β were analyzed next. Compared to the transcriptome of LIF + 2i, the WT transcriptome lacking 2i (WT LIF only) exhibited the largest number of DEGs, thus manifesting the most severe alterations. Therefore, to compare the effect of depletion of each inhibitor, ‘WT LIF only’ DEGs were designated as ‘gene sets for 2i’ for comparison. Within the ‘gene sets for 2i’, KD naïve ESCs lacking iMek1 [KD (-) iMek1] were altered the least (Fig. 2H) compared to the other cells, suggesting that KD naïve ESCs would be more tolerant to iMek1 depletion.

As predicted, unlike WT cells, the ‘colonial dome shape’ morphology of KD naïve ESCs remained unaltered without iMek1 supplementation but was quickly lost after iGsk3β withdrawal (Fig. 2I). Given that Shp2 has another role as a signal transducer to Ras-to-Erk in addition to tyrosine phosphatase, a lack of Shp2 may decouple the signal transduction to Erk upon LIF stimulation. Consistent with the results in Fig. 2G and H, phosphorylated Mek1 and Erk (Additional file 1:Fig. S2F) was attenuated in KD naïve ESCs, whereas no clear alteration in GSK3β phosphorylation was observed (Additional file 1:Fig. S2G). These data were also supported by GSEA, showing that the gene signatures of “Hallmark KRAS signaling DN and UP” were more enriched in KD naïve ESCs when iMek1 was depleted (Additional file 1:Fig. S2H).

### Role of Shp2 in primed pluripotency

Unlike naïve ESCs, primed ESCs were likely intolerant to the absence of Shp2. Despite multiple trials, the establishment of primed ESCs with stable Shp2 depletion was unsuccessful due to the severe growth retardation of primed ESCs after Shp2 knockdown (Additional file 1:Fig. S3). Alternatively, KD naïve ESCs were subjected to primed culture conditions (with bFGF/Activin) to establish KD primed-like ESCs. Although KD naïve ESCs propagated well in naïve culture conditions, they did not successfully grow in primed culture conditions (Fig. 3A and Additional file 10: Movie S3A, Additional file 11: Movie S3B, Additional file 12: Movie S3C, Additional file 13: Movie S3D). This dichotomous effect of Shp2 depletion on primed ESCs compared to naïve ESCs was further highlighted by clonogenic assays (Fig. 3B), which account for prompt culling of KD ESCs (labeled with GFP only) from the WT ESCs (labeled with RF/GFP) mixture under primed conditions unlike naïve conditions (Fig. 3C and Additional file 14: Movie S4A, Additional file 15: Movie S4B). As similar as that of naïve ESCs, signaling to Erk upon bFGF/Activin stimulation was markedly attenuated in KD ESCs (Fig. 3D). These results indicate that the self-renewal of primed ESCs depends on Shp2-dependent signaling unlike that of naïve ESCs.

### Defects in the differentiation of naïve ESCs by Shp2 depletion

**Fig. 4:**
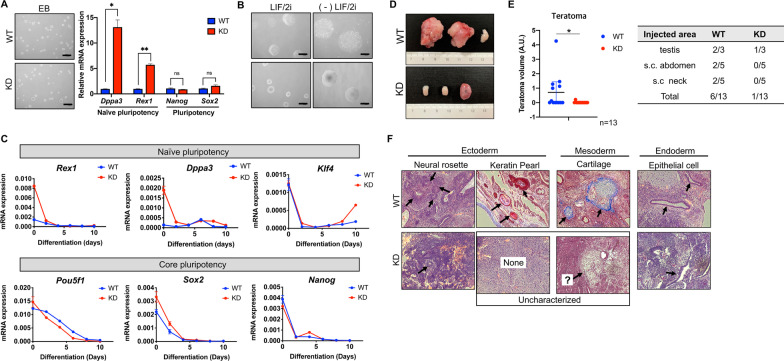
Defects in the differentiation of naïve ESCs by Shp2 depletion. **A** Light microscopic images of embryonic body (EB) cultured for 3 days of WT or KD ESCs (scale bars, 500 μm) (left), Graphical presentation of relative mRNA expression of typical naïve pluripotency and core pluripotency markers (right) of WT (blue) and KD (red). **B** Light microscopic images of WT and KD before [LIF/2i] or 48 h after differentiation [(-)LIF/2i].**C** Relative mRNA expressions of naïve pluripotency (top) and core pluripotency (bottom) markers at indicative time during spontaneous differentiation of WT (blue) and KD (red). **D** Representative images of teratomas formed in mouse testes at 6 weeks after injection of WT (top) and KD (bottom) ESCs. **E** Graphical presentation of teratoma volume [*V* = *a* × *b* × *c* × π/6 (*a* = length, *b* = width, and *c* = depth] of total 13 teratoma from WT or KD ESCs (left), formed three different areas (right), volumes of normal testes after teratoma injection were considered as 0. **F** Hematoxylin & eosin (H&E) (for ectoderm and endoderm) and Masson’s trichrome (for mesoderm) staining of teratoma section from WT and KD, black arrows represent typical tissues for ectoderm, mesoderm and endoderm. Uncharacterized tissue from teratoma of KD were shown in the box

Shp2 null mice failed to develop as a result of peri-implantation lethality, which is caused by trophoblast failure [37]. Similar to the high bFGF signaling dependence described above, trophoblast stem cells (TS) and epiblast stem cells (EpiSCs), which share a close similarity to primed ESCs, rely on bFGF signaling [38]. As previously proposed [37], embryo development failure in Shp2 null mice may result from defects in the transition from naïve to primed ESCs in accordance with the TS impediment. Naïve ESCs after Shp2 depletion tended not only to maintain naïve pluripotency (Fig. 2) but also to hinder the progression to primed ESCs (Fig. 3), and therefore the differentiation potential of KD naïve ESCs would be disturbed. Thus, WT and KD naïve ESCs were subjected to spontaneous differentiation via LIF/2i withdrawal from embryoid body (EB) formation (Fig. 4A). During spontaneous differentiation, which initiates with EB formation, typical marker genes of naïve pluripotency were clearly upregulated in the embryoid bodies of KD naïve ESCs (Fig. 4A). Along with different EB morphology (Fig. 4B), naïve pluripotency genes remained marginally high one day after differentiation induction and sharply suppressed when core pluripotency marker genes were similarly repressed (Fig. 4C). Thus, spontaneous differentiation was enforced with the serum-induced exit from naïve pluripotency regardless of Shp2. However, unlike in vitro differentiation, the formation of teratoma from KD naïve ESCs was significantly impaired in multiple sites compared to those from WT (Fig. 4D). One teratoma-like mass that was formed out of a total of 13 injections of KD naïve ESCs (Fig. 4E) only exhibited a few ectoderm and endoderm tissue structures without clear mesoderm tissue formation, unlike the well-developed teratoma from WT (Fig. 4F). It is also worth noting that Shp2 is required for proper gastrulation and mesoderm patterning in mouse [39] and *Xenopus* [40] development.

### Shp2 chemical inhibitor as an iMek1 replacement

**Fig. 5:**
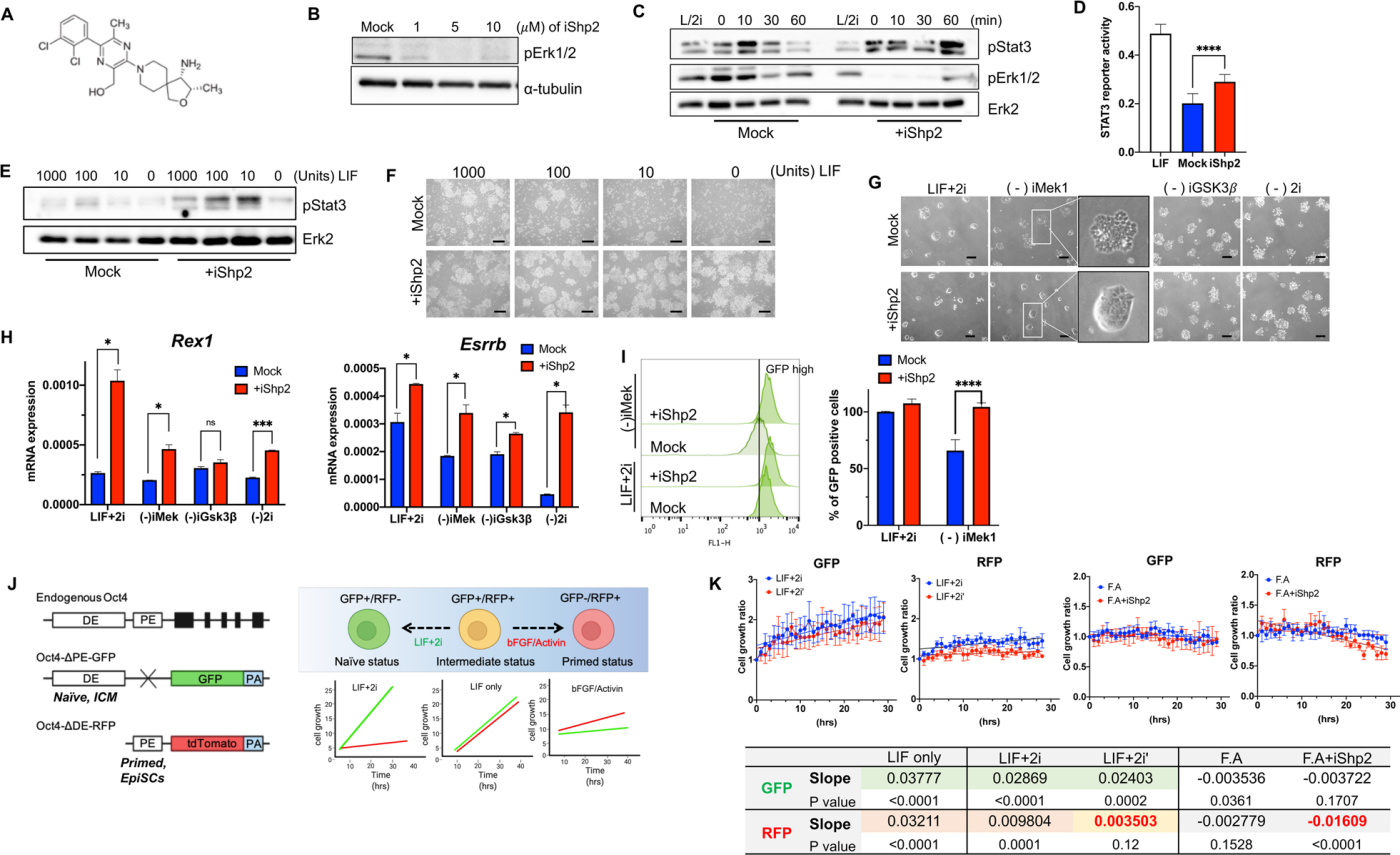
Shp2 chemical inhibitor as an iMek1 replacement. **A** Chemical structure of Shp2 inhibitor (iShp2: RMC-4550). **B** Immunoblotting analysis of WT ESCs at 30 min after indicative dose of iShp2 treatment, α-tubulin was used as a loading control. **C** Immunoblotting analysis of WT ESCs at indicted time after 1000 units of LIF stimulation, pretreated with either DMSO (Mock) or 5 μm of iShp2 (+ iShp2) for 1 h while LIF starvation (L/2i: LIF + 2i control). **D** Graphical presentation of luciferase reporter activity of Stat3 at 8 h after LIF deprivation with either DMSO (Mock) or 5 μm of iShp2 (+ iShp2) for 1 h prior to LIF deprivation. White column (LIF) indicates reporter activity of Stat3 without LIF deprivation as a control (***p < 0.001, *n* = 3). **E** Immunoblotting analysis for pStat3 at 30 min after indicative dose of LIF stimulation in WT ESCs pretreated with either DMSO (Mock) or 5 μm of iShp2 (+ iShp2) for 1 h while LIF starvation. **F** Light microscopic images of WT cultured under indicative dose of LIF for 3 days supplemented with DMSO (Mock) or 5 μm of iShp2 (+ iShp2) (scale bars, 500 μm). **G** Light microscopic images of WT ESCs under control (LIF + 2i), deprivation of iMek1 [(-)iMek1], iGsk3β [(-)iGsk3β] or iMek1/iGsk3β[(-2i)] with DMSO (Mock) or 5 μm of iShp2 (+ iShp2) treatment for 2 days (scale bars, 200 μm) **H** Relative gene expression of *Rex1* (left) and *Essrb* (right) at indicated culture condition with DMSO (Mock: blue) or 5 µM of iShp2 (+ iShp2: red) for 2 days (*p < 0.05, **p < 0.01, ***p < 0.001, n.s. for not significant). **I** Flow cytometry of GFP intensity of WT ESCs with either LIF + 2i control (LIF + 2i) or iMek1 depletion [(-)iMek1] for 2 days, pretreated with DMSO (Mock) or 5 μM of iShp2 (+ iShp2) (****p < 0.0001, *n* = 6). **J** Graphical illustration of endogenous Oct4 with distal enhancer (DE) and proximal enhancer (PE)(top), Oct4-ΔPE-GFP (middle) and Oct4-ΔDE-RFP (bottom), activation of DE and PE at naïve and inner cell mass (ICM) and at primed and epiblast stem cells (EpiSCs) respectively (left), ESCs with GFP + /RFP- (Green), GFP + /RFP + (Yellow) and GFP-/RFP + (red) at naïve, intermediate and primed status respectively (top), expected growth of GFP + or RFP + ESCs under each indicative condition was shown (bottom). **K** Cell growth ratio at indicated time of GFP + or RFP + ESCs under LIF + 2i/LIF + 2i’ (iShp2 instead of iMek1) and bFGF/Activin with DMSO (F.A) or iShp2 (F.A + iShp2) (top panels), Table of slope and *p* value of linear regression of GFP + or RFP + at indicated culture condition, effect of iShp2 on slope was highlighted in red

There is emerging evidence that Shp2 contributes to chemoresistance and cancer development [21, 22, 41], and therefore Shp2 allosteric inhibitors that interfere in both phosphatase and signal transduction have been developed as novel anti-cancer therapeutic agents [21]. We first examined whether an allosteric Shp2 inhibitor (Fig. 5A, RMC-4550: iShp2), which was initially developed to decouple the oncogenic Ras-to-Erk signaling in human cancers [21], inhibits LIF-dependent Erk activation. At an iShp2 concentration known to decrease the basal level of Erk2 phosphorylation (Fig. 5B), Stat3 phosphorylation was significantly sustained after LIF stimulation in naïve ESCs (Fig. 5C), which was associated with higher Stat3 reporter activity in the presence of iShp2 8 h after LIF deprivation (Fig. 5D). Additionally, iShp2 treatment also preserved Stat3 phosphorylation even at a 1/100-fold LIF concentration (Fig. 5E) and markedly rescued naïve ESCs from cell death at a low LIF concentration (Fig. 5F). Similar to our observations in KD naïve ESCs (Fig. 2I), iShp2 treatment could likely replace the effects of iMek1 (but not iGSK3β) on the ‘colonial dome shape’ morphology of naïve ESCs (Fig. 5G), which may result from the clear decoupling of LIF mediated Ras-to-Erk signaling by iShp2 treatment (Fig. 5C). Similarly, typical naïve marker genes (Fig. 5H) and the GFP signal (Fig. 5I) indicated that iShp2 treatment compensated for the loss of iMek1 in naïve ESCs. Next to validate the dichotomous effect of iShp2 in naïve and primed ESCs, we took advantage of mESCs expressing GFP and/or RFP due to distinct enhancer activity of Pou5f1 (encoding Oct4) in naïve (or ICM) [under control of distal enhancer (DE)] and primed (or epiblast) [under control of proximal enhancer (PE)] ESCs [42] (Fig. 5J). As illustrated in Fig. 5J, while ESCs of intermediate status expressing both GFP and RFP proliferate under LIF only condition (Fig. 5J), naïve (e.g., GFP + only) and primed (e.g., RFP + only) ESCs would exclusively expand under LIF + 2i and bFGF/Activin culture condition respectively (Fig. 5J). As expected, both GFP and RFP signal from intermediate ESCs was gradually increased under ‘LIF only’ condition (Additional file 1:Fig. S4A). To contrast, GFP but not RFP signal became readily dominant under LIF + 2i while RFP signal was only marginally affected by bFGF/Activin culture (Additional file 1:Fig. S4B and C). As conversion from the intermediate status to primed ESCs (expressing only RFP +) requires multiple passaging as described previously [42], RFP as well as GFP signal just barely maintained by bFGF/Activin. Of note, iShp2 treatment was likely to interfere in the increase of RFP rather than GFP signal under LIF + 2i (vs. LIF + 2i’) and bFGF/Activin (vs F.A + iShp2) (Fig. 5K, Additional file 16: Movie S5A, Additional file 17: Movie S5B, Additional file 18: Movie S5C, Additional file 19: Movie S5D, Additional file 20: Movie S5E), implying that Shp2 inhibition would be unfavorable for ESCs that are under control PE of *Pou5f1*.

## Discussion

The disrupted activity of Shp1 [2] and 2 [26] in mESCs due to a lack of Zap70, a protein that acts as a non-receptor tyrosine kinase upon LIF stimulation, increases Jak/Stat3 signaling and self-renewal. However, although we observed that the effect of Zap70 depletion on hESCs was only marginal unlike mESCs (data not shown), we simply assumed that these discrepancies were largely attributable to species-specific factors and other inherent differences [1]. However, recent advances in the characterization of naïve and primed pluripotency have now revealed that such dissimilarities between mouse and human ESCs result from the unique characteristics of naïve and primed pluripotent stem cells.

Unlike in primed ESCs, the simultaneous chemical inhibition of Mek1 and Gsk3β is critical for the maintenance of naïve pluripotency [9], thus highlighting the unique cellular signaling in the ICM of blastocysts [12, 43]. Particularly, the normal development of Erk2 null embryos until pre-implantation [44] and low basal Erk activity in ICM [13] suggest that Erk activity is dispensable during the pre-implantation state, which is consistent with the characteristics of naïve pluripotency. Similarly, Shp2 null embryo lethality also results from trophoblast failure [37] and possible epiblast cell death with primed characteristics. To sharp contrast, embryos lacking *Socs3*, another negative regulator of Jak/Stat signaling like Shp2, develop normally till E10.5 and die due to placental abnormality [45], implying marginal effect on early embryogenesis.

The requirement of DUSPs, Erk1/2 specific phosphatases, in mESCs also demonstrates that Erk activity needs to be maintained at a lower level to hold naïve pluripotency [14, 15]. Nevertheless, the constant requirement of the Mek1 inhibitor during in vitro culture of naïve ESCs implies that constant Erk activation occurs due to LIF stimulation, which eventually impedes naïve pluripotency via Erk-dependent phosphorylation [46, 47].

Meta-analysis of previous datasets from a genome-wide study [31] revealed that Shp2 was predicted to serve as a negative regulator for naïve pluripotency through its involvement in both LIF-Jak/Stat3 and Ras-to-Erk signaling (Fig. 1). Notably, Shp2, whose catalytic activity was induced by LIF along with tyrosyl phosphorylation [18], serves not only as a negative regulator for Stat3 but also as a positive regulator for Ras-to-Erk (Fig. 2). This is why the decoupling of ‘Ras-to-Erk’ signaling occurred through Shp2 inhibition, thus liberating the demand of iMek1 for naïve pluripotency (Fig. 5) as well as enhancing naïve pluripotency (Fig. 2). In sharp contrast, Shp2 appeared to be indispensable for primed ESCs, whose pluripotency relies on bFGF/Activin stimuli. Perturbation of Shp2 significantly delayed the self-renewal of ESCs in primed conditions (Figs. 3 and 5J) with concurrent decoupling of Erk signaling (Fig. 3D). Additionally, Shp2 suppression can successfully substitute the usage of iMek1. Therefore, the interfering dual roles of Shp2 with the allosteric inhibitor of Shp2 [21] used in this study prolonged Stat3 activation and attenuated Erk phosphorylation, which mimicked the effect of Shp2 depletion on naïve pluripotency (Fig. 5).

Recently, prolonged usage of iMek1 was demonstrated to cause irreversible changes of (epi)genome, affecting developmental potential [48], which triggers the massive chemical screening for alternatives [49]. Considering the key roles of Shp2 for Erk signaling [16] and successful substitute for iMek1 (Fig. 5), iShp2 would be able to serve as the potential alternative for iMek1 once the effect on status of (epi)genome is determined.

## Conclusions

Shp2 serves as a negative regulator for naïve pluripotency due to its dual roles in Jak/Stat3 and Ras-to-Erk signaling, whereas the pluripotency of primed cells depended on bFGF/Activin, which could also be modulated by Shp2-dependent signaling. Allosteric inhibition of Shp2 with an inhibitor to disrupt these dual functions can therefore be used to improve naïve pluripotency and replace the use of iMek1. Considering the complex protocol for human naïve pluripotent stem cell conversion [50], iShp2 treatment would be a promising strategy for the efficient establishment of human naïve PSCs, which will be examined in future studies.

## Materials and methods

### Reagents

The primary antibodies against α-tubulin (#sc-8035), β-actin (#sc-47778), Stat3 (#sc-8019), Erk2 (#sc-154) were purchased from Santa Cruz Biotechnology, Inc. Antibodies against phospho-Shp2 (Tyr542) (#3751), phospho-Stat3 (#9145s), phospho-Erk1/2 (#9101), phospho-Mek1/2 (#9121s) were purchased from Cell Signaling Technology. Antibodies against Shp2 (#PA5-95069) and phospho-Src (#44660 g) were purchased from Invitrogen. The secondary antibodies against mouse IgG (#115-035-003) and rabbit IgG (#111-035-003) were purchased from Jackson ImmunoResearch Laboratories, Inc. siRNAs targeting Negative Control (#SN-1003) and the others were obtained from Bioneer. Expression vectors of 6 × Stat3-luciferase was kindly gifted by Prof. Hyewon Youn from Seoul National University.

### Cell culture

Naïve mouse ESCs were cultured on 0.5% porcine gelatin-coated dish in Naïve mESC culture media -DMEM high glucose (Gibco) supplemented with 15% FBS (Gibco), 1% Glutamax (Gibco), 1% MEM-nonessential amino acids (Gibco), 0.1% Gentamycin (Gibco), 0.1 mM β-mercaptoethanol (Gibco), 1,000 U/ml mouse leukemia inhibitory factor (mLIF) (Millipore, Merck), 1 μm PD0325901 (Peprotech) and 3 μm CHIR99021- at 37 °C and 5% CO_2_ incubating condition. Cells were passaged 1:20 ratio every 3 days using 0.25% Trypsin/EDTA (Wellgene) as a single cell dissociation reagent. Primed mouse ESCs were cultured on Matrigel (Corning# 354277)-coated dish in EpiSC culture media -DMEM/F12 (Gibco) supplemented with 15% KnockOut SR (Gibco), 1% GlutaMAX (Gibco), 1% MEM-nonessential amino acids (Gibco), 0.1% Gentamycin (Gibco), 10 ng/ml murine bFgf (Peprotech), 20 ng/ml murine Activin (Peprotech)- at 37 °C and 5% CO_2_ condition. Primed mESCs were passaged every 3–4 days using Dispase (Gibco) as a colony detachment reagent. Detached colony clumps were transferred 1:15–1:20 ratio on Matrigel (Corning# 354277) coated dishes. Culture media was changed every day for all cell lines. Oct4-ΔPE-GFP/ΔDE-RFP ESCs were cultured on mitogen-inactivated C57BL/6 feeder cells on 0.15% porcine gelatin-coated dish in culture media-DMEM Low (Gibco) supplemented with 15% FBS (Gibco), 1% P.S.G (Gibco), 1% MEM-nonessential amino acids (Gibco), 0.1 mM β-mercaptoethanol (Gibco) and 1000 U/ml mouse leukemia inhibitory factor (mLIF) (Millipore, Merck)- at 37 °C and 5% CO_2_ incubating condition.

### Teratoma formation assay

5 × 10^5^ cells of each OG2 and OG2Shp2KD mESCs were injected into the testes of 5-weeks-old male BALB/C nude mice (*n* = 3), and subcutaneously injected in the neck (*n* = 5) and abdomen (*n* = 5) of 5-weeks-old male BALB/C nude mice. Mice were euthanized 6 weeks after the injection. Animal experiments were conducted under the permission of Seoul National University Institutional Animal Care and Use Committee (Permission number: SNU-190716–5-2).

### RNA-sequencing analysis

Total RNA was isolated from cell pellets by using Easy-BLUE™ RNA isolation kit (iNtRON Biotechnology). 1 mg of total RNA was processed for preparing mRNA sequencing library using MGI Easy RNA Directional Library Prep Kit (MGI) following manufacturer’s instruction. The mRNA is fragmented into small pieces under elevated temperature. The cleaved RNA fragments are copied into first strand cDNA using reverse transcriptase and random primers. Strand specificity is achieved in the RT directional buffer and second strand cDNA synthesis was done. Single ‘A’ base was added to the cDNA fragments, followed by ligation of the adapter. The products are purified and enriched by PCR procedure to acquire the final cDNA library. The cDNA library is quantified using QuantiFluor ONE dsDNA System (Promega). Using DNA nanoball (DNB) enzyme, the library is incubated at 30 °C for 25 min to make DNB. Finally, sequencing of the prepared DNB was performed using MGIseq system (MGI) with 100 bp paired-end reads.

### Establishment of Shp2KD cell line

Shp2 stable knocked-down cell line was acquired by introducing shPtpn11 Piggy-Bac vector and Transposase vector. Transfection was performed using Lipofectamine 3000 reagent (#L3000-001, Invitrogen). G418 selection (600 μg/ml) was done for 9 days. Single colony selection was performed from heterogenous Shp2KD pool cells.

### Flow cytometric analysis

GFP intensity of OG2 cells were measured by Flow cytometric analysis. Cells were dissociated with Accutase (#561527, BD Bio-sciences) and washed three times with DPBS. Cells were analyzed through FACS Carlibur (BD Bioscience). GFP intensity was determined by measuring FITC channel.

### Live cell imaging

Brightfield images and GFP images of cells on specific area were captured every hour by JuLi Stage (NanoEntek). Acquired images were merged to create time-lapse cell history movies using JuLi-Edit. Relative fluorescence intensity and area of acquired images were measured by using the ImageJ analysis program (ImageJ bundled with ZuluOpen JDK 13.0.6 version, National Institutes of Health, Bethesda, MD, USA; https://imagej.nih.gov/ij).

### Cell tracing

Cell tracing was performed with CellTrace™ Far Red Cell Proliferation Kit (Invitrogen). Cells were pre-treated with CellTrace™ Far Red reagent (1:1000) for 24 h. Both Far Red-labeled and unlabeled cells were mixed 1:1 ratio and co-cultured. Fluorescence images of live cell was acquired by JuLi Stage (NanoEntek).

### RNA isolation and quantitative RT-PCR analysis

Total RNA was isolated from cells using Easy-Blue™ total RNA isolation kit (iNtRON Biotech) following the manufacturer’s instructions. 5 × PrimeScript™ RT mix (TaKaRa) was used during reverse transcription to acquire cDNA. Quantitative real-time PCR was performed using 2 × TB-Green premix (TaKaRa) by LightCycler-480^®^II (Roche). *Rn18s* gene was used as internal loading control for normalizing gene expression data (Table 1).

**Table 1:** Sequences of primers for quantitative real-time PCR

Primer pairs		Primer sequences (5’-3’)
Ptpn11	Forward	AGTCCAAAGTGACCCACGTC
	Reverse	CCATCATGCAGAACGACCCT
Dppa3	Forward	CGTACCTGTGGAGAACAAGAGTG
	Reverse	CA TTCTCAGAGGGA TCCCA TCTTTG
Rex1	Forward	CTTCGAAAGCTTGGAGGAAGTGGAG
	Reverse	GGACACTCCAGCATCGATAAGACAC
Nr0b1	Forward	ACAGAGCAGCCACAGA TGGTGTC
	Reverse	GATGTGCTCAGTAAGGATCTGCTG
Klf4	Forward	GAACAGCCACCCACACTTGTGAC
	Reverse	CTGTCACACTTCTGGCACTGAAAG
Esrrb	Forward	GATTCTCATCTTGGGCATCGTGTAC
	Reverse	CTGACTCAGCTCATAGTCCTGCAG
Klf2	Forward	CACACATACTTGCAGCTACACCAAC
	Reverse	CAAGTGGCACTGAAAGGGTCTGTG
Fgf5	Forward	CATCGGTTTCCATCTGCAGATCTAC
	Reverse	GTTCTGTGGATCGCGGACGCATAG
Cer1	Forward	GTGGAAAGCGA TCA TGTCTCA TCG
	Reverse	GCAAAGGTTGTTCTGGACAACGAC
Pou5f1	Forward	GAGAAAGCGAACTAGCATTGAGAAC
	Reverse	TGTAGCCTCATACTCTTCTCGTTG
Sox2	Forward	ATGGGCTCTGTGGTCAAGTC
	Reverse	CCCTCCCAATTCCCTTGTAT
Nanog	Forward	GTGCACTCAAGGACAGGTTTCAG
	Reverse	CTGCAATGGATGCTGGGATACTC
c-Myc	Forward	ACCACCAGCAGCGACTCTGA
	Reverse	TGCCTCTTCTCCACAGACACC

### Dual luciferase reporter assay

Cells were co-transfected with 6×Stat3 reporter vector and pRL vector using Lipofectamine 2000 reagent (#11668019, Invitrogen). After 3 h of incubation with 1 × passive lysis buffer followed by centrifugation, supernatant from the cell lysate was acquired. The supernatant was used for the reaction with LARII and Stop & Glo reagent. Stat3 luciferase assay was performed using Dual Luciferase Reporter Assay System Kit (#E1980, Promega), and detected by SpectraMax^®^ i3x Multi-Mode Microplate Reader.

### Phosphatase activity assay

Shp2 phosphatase activity was carried out with PTP assay kit 1 (#17-125, Sigma-Aldrich) according to the manufacturer’s protocol. In brief, 10 mM of p-nitrophenylphosphate (pNPP), used as the substrate for phosphatase activity assay was incubated at 30 °C for 30 min under assay buffer (30 mM HEPES at pH 7.4, 120 mM NaCl, 5 mM DTT). The relative optical density was determined by 405 nm absorbance using Epoch (BioTek).

### Immunoblotting analysis

Whole Cell lysate was extracted using RIPA buffer (Biosesang), supplemented with 1 μM protease inhibitor, 10 μM sodium orthovanadate. After 1 h incubation on ice followed by centrifugation, whole cell lysate was acquired. Quantification of proteins were performed with pierce BCA protein assay Kit (#23225, Thermo Fischer Scientific). Protein sample was prepared by adding 5 × SDS-PAGE loading buffer (#SF2088-110-00, Biosesang) and boiling at 100 °C for 10 min. Approximately 15 μg of each total protein was loaded and separated on 10% SDS-PAGE gel. Separated proteins were transferred to activated PVDF membrane. Membrane with transferred proteins was blocked with 5% skim milk in TBS-T in RT for 1 h followed by washing. Primary antibody (1:500 ~ 1:1000) in TBS-T was incubated with 1% sodium azide at 4 °C for overnight. After washing, the membrane was incubated with secondary antibody (1:10000) in TBS-T at RT for 1 h. Chemoluminescence was detected by Chemi-Doc using Miracle-Star (#16028, iNtRON Biotechnology) kit or West-Queen (#16026, iNtRON Biotechnology) kit.

### Immunoprecipitation

Cell lysate was extracted using RIPA buffer (Biosesang), (1 μM protease inhibitor, 10 μM sodium orthovanadate). After 1 h incubation on ice followed by centrifugation, whole cell lysate was acquired. 1 μg of Shp2 antibody (#VL3159027A, Invitrogen) was added in protein lysate and incubated at 4 °C for overnight. Protein A agarose (#sc-2001, Santa-cruz) was added in protein lysate and incubated at 4 °C for 4 h. Beads were gathered by centrifugation and protein sample was prepared by adding 5 × SDS-PAGE loading buffer (#SF2088-110-00, Biosesang), followed by boiling at 100 °C for 10 min.

### Gene set enrichment analysis (GSEA)

FPKM values of RNA sequencing data were formatted and loaded to run Gene Set Enrichment Analysis. GSEA software and the Gene Sets (Hallmark_IL6_JAK_STAT3 signaling, KEGG_JAK_STAT3 signaling, ABBUD_LIF signaling_up, Hallmark_KRAS_signaling_DN, Hallmark_KRAS_signaling_UP) were downloaded from Molecular Signatures Database v7.4 (https://www.gsea-msigdb.org).

## Statistical analysis

The quantitative data are presented as the mean values ± standard deviation (SD). Unpaired two-tailed *t*-tests or one-way ANOVA following Dunnett multiple comparison, was performed to analyze the statistical significance of each response variable. *p*-values less than 0.05 were considered statistically significant (*< 0.05, **< 0.01, *** < 0.001, **** < 0.0001 and n.s. for not significant).

## Supplementary Information

**Additional file 1:** Supplementary Figures 1-4, Supplementary Figure legends, Supplementary Movie legends.

**Additional file 2: Movie S1A.** Fluorescence microscopic live images of WT ESCs under LIF+2i condition (Figure S1A).

**Additional file 3: Movie S1B.** Fluorescence microscopic live images of WT ESCs under LIF only condition (Figure S1A).

**Additional file 4: Movie S1C.** Fluorescence microscopic live images of WT ESCs under LIF- condition (Figure S1A).

**Additional file 5: Movie S2A.** Fluorescence microscopic live images of WT ESCs under LIF+2i condition (Figure 2D).

**Additional file 6: Movie S2B.** Fluorescence microscopic live images of KD ESCs under LIF+2i condition (Figure 2D).

**Additional file 7: Movie S2C.** Fluorescence microscopic live images of WT ESCs under LIF only condition (Figure 2D).

**Additional file 8: Movie S2D.** Fluorescence microscopic live images of KD ESCs under LIF only condition (Figure 2D).

**Additional file 9: Table S1.** Gene lists of regulators for naïve pluripotency (Figure S1C).

**Additional file 10: Movie S3A.** Light microscopic live images of WT under LIF+2i condition (Figure 3A).

**Additional file 11: Movie S3B.** Light microscopic live images of WT under bFGF/Activin condition (Figure 3A).

**Additional file 12: Movie S3C.** Light microscopic live images of KD under LIF+2i condition (Figure 3A).

**Additional file 13: Movie S3D.** Light microscopic live images of KD under bFGF/Activin condition (Figure 3A).

**Additional file 14: Movie S4A.** Fluorescence microscopic live images of co-cultured WT and KD ESCs under LIF+2i condition (Figure 3C).

**Additional file 15: Movie S4B.** Fluorescence microscopic live images of co-cultured WT and KD ESCs under bFGF/Activin condition (Figure 3C).

**Additional file 16: Movie S5A.** Fluorescence microscopic live images of GFP and RFP under LIF only condition (Figure S4A).

**Additional file 17: Movie S5B.** Fluorescence microscopic live images of GFP and RFP under LIF+2i condition (Figure S4B, S4C).

**Additional file 18: Movie S5C.** Fluorescence microscopic live images of GFP and RFP under LIF+2i' condition (Figure S4B, S4C).

**Additional file 19: Movie S5D.** Fluorescence microscopic live images of GFP and RFP under bFGF/Activin condition (Figure S4B, S4C).

**Additional file 20: Movie S5E.** Fluorescence microscopic live images of GFP and RFP under bFGF/Activin +iShp2 condition (Figure S4B, S4C).

## Data Availability

Source data are available from the corresponding authors upon request.
