# Supplementary material for: Dichotomous role of Shp2 for naïve and primed pluripotency maintenance in embryonic stem cells
Source: Stem Cell Res Ther. 2022 Jul 18;13:329. doi: 10.1186/s13287-022-02976-z (PMC9290224; doi:10.1186/s13287-022-02976-z)
Supplement: Supplementary file 1 — Additional file 1: Supplementary Figures 1-4, Supplementary Figure legends, Supplementary Movie legends. [file 13287_2022_2976_MOESM1_ESM.pdf]

Supplementary Information

Dichotomous role of Shp2 for naïve and primed pluripotency maintenance in embryonic stem cells

Seong-Min Kim, Eun-Ji Kwon, Yun-Jeong Kim, Young-Hyun Go, Jeong-Tae Do,

Keun-Tae Kim and Hyuk-Jin Cha

This PDF file includes:

Supplementary Figure legends

Supplementary Movie legends

Supplementary Figures 1-4

Supplementary Figure legends

Figure S1. (A) Fluorescent microscopic images at indicated time under LIF+2i, LIF only and LIF deprivation (LIF-) media respectively (scale bars : 500μm). (B) Luciferase reporter activity of Stat3 in OG2 cells at indicated time after LIF stimulation (*, $p<0.05$, **, $p<0.001$, ***, $p<0.0001$, n.s. for not significant). (C) Table of KEGG pathway enriched by genes from putative negative (27) and positive (128) regulators for naïve pluripotency. (D) Top enriched categories of GO from BioPlanet 2019, Wiki Pathways 2019 Mouse, and MSigDB Hallmark 2020 of putative negative (27) and positive (128) regulators for naïve pluripotency, ranked by -log(p-value). (E, F, G) KEGG pathway for Signaling Pathways regulating Pluripotency of Stem Cells (E), Jak/Stat signaling pathway (F), and Ras signaling pathway (G) in Mus musculus. 27 and 128 putative negative and positive regulators for naïve pluripotency were shown in red and blue respectively.

Figure S2. (A and B) Relative mRNA expressions of naïve pluripotency markers (A) and primed makers (B) in OG2 (green) and POG2 (grey) ESCs (***, $p < 0.0001$ and ****, $p < 0.00001$, n=3). (C) Fold mRNA expressions of Ptpn11 in WT (blue) and KD (red) ESCs (*, $p < 0.05$). (D) Fold mRNA expressions of core pluripotency markers in WT (blue) and KD (red) ESCs (n.s., non-significant). (E) Light microscopic images of WT and KD ESCs under indicated media conditions (scale bars, 500μm). (F) Immunoblotting analysis for Shp2, pMek1/2, Erk2 and α-tubulin in WT and KD ESCs. (G) Immunoblotting analysis for pGsk3β, pErk1/2, pStat3 and Vinculin of WT and KD ESCs at indicated time after LIF stimulation. Vinculin was used as a loading control. LIF starvation for 1hour was performed before the LIF stimulation. (H) The normalized enrichment score by gene set enrichment analysis (GSEA) of WT and KD ESCs for HALLMARK_KRAS_SIGNALING_DN (left) and HALLMARK_KRAS_SIGNALING_UP (right).

Figure S3. Light microscopic images of POG2-WT and KD cells (scale bars, 500μm).

Figure S4. (A) Cell growth ratio of GFP+ and RFP+ ESCs under LIF only culture condition media (left) and fluorescence microscopic images of GFP+ and RFP+ at indicated time (scale bars, 500μm) (right) (B and C) Fluorescence microscopic images of GFP under control of distal enhancer (DE-GFP) and RFP under control of proximal enhancer (PE-RFP) of GO2 ESCs under indicated media conditions (scale bars, 500μm).

Supplementary Movie legends

Movie S1. Fluorescence microscopic live images of cell growth of WT ESCs under LIF+2i (A), LIF only (B) and LIF deprivation condition (C) (scale bars = 250μm).

Movie S2. Fluorescence microscopic live images of cell growth of WT (A) and KD (B) under LIF+2i and LIF only (C: for WT, D for KD) condition (scale bars = 250μm).

Movie S3. Light microscopic live images of cell growth of WT under LIF+2i (A) and bFGF/Activin (B) and KD under LIF+2i (C) and bFGF/Activin (D) (scale bars = 500μm).

Movie S4. Fluorescence microscopic live images of co-cultured red fluorescence and GFP labeled WT and GFP only labeled KD ESCs under LIF+2i (A) and bFGF/Activin (B) culture condition (scale bars = 500μm).

Movie S5. Fluorescence microscopic live images of green and red fluorescence under LIF only (A), LIF+2i (B), LIF+2i' (iShp2 instead of iMek1) (C), bFGF/Activin (D) and bFGF/Activin supplemented with iShp2 (E) culture condition (scale bars = 500μm).

Figure S1

A

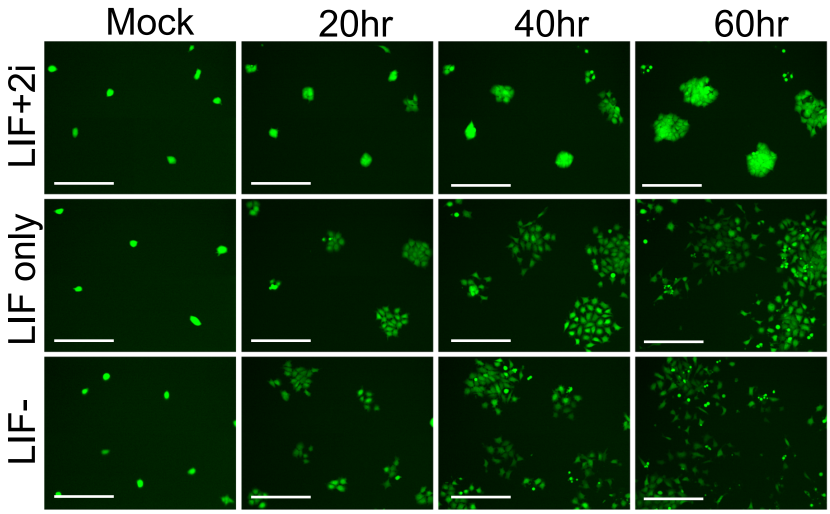

B

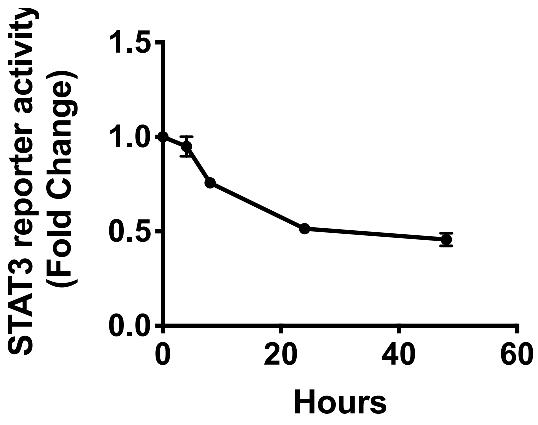

C

GFP+:GFP- Positive Hit Genes (27)	GFP+:GFP- Negative Hit Genes (128)
Putative negative regulators for naïve pluripotency	Putative positive regulator for naïve pluripotency
Pathway in Cancer, Signaling pathways regulating pluripotency, JAK/STAT signaling, RAS signaling	

D

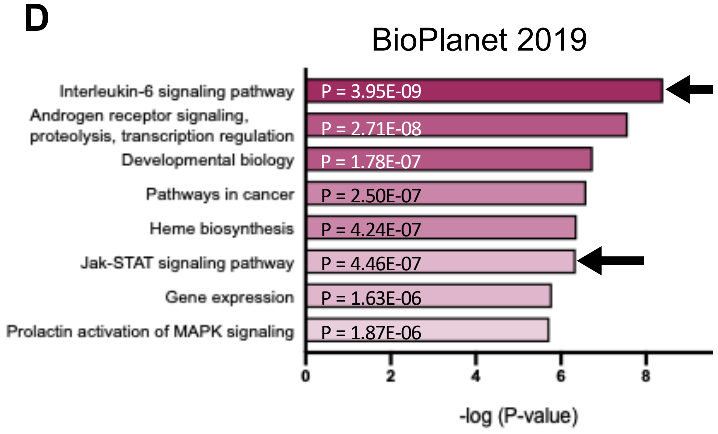

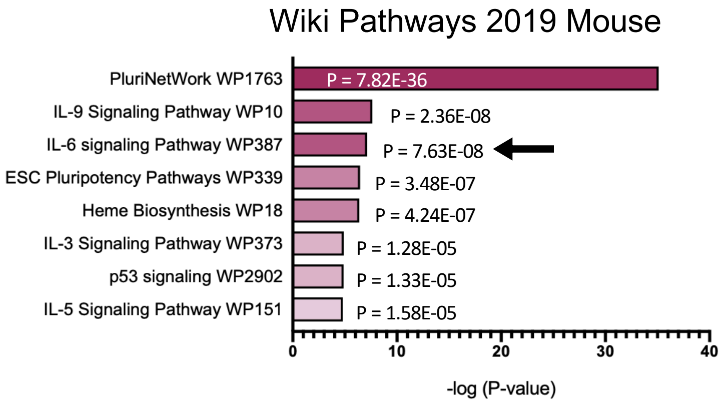

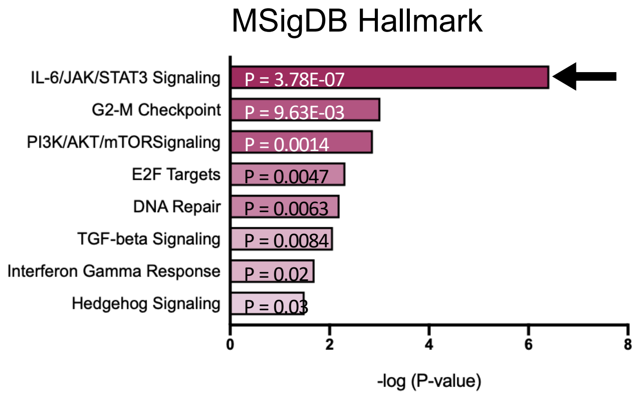

E

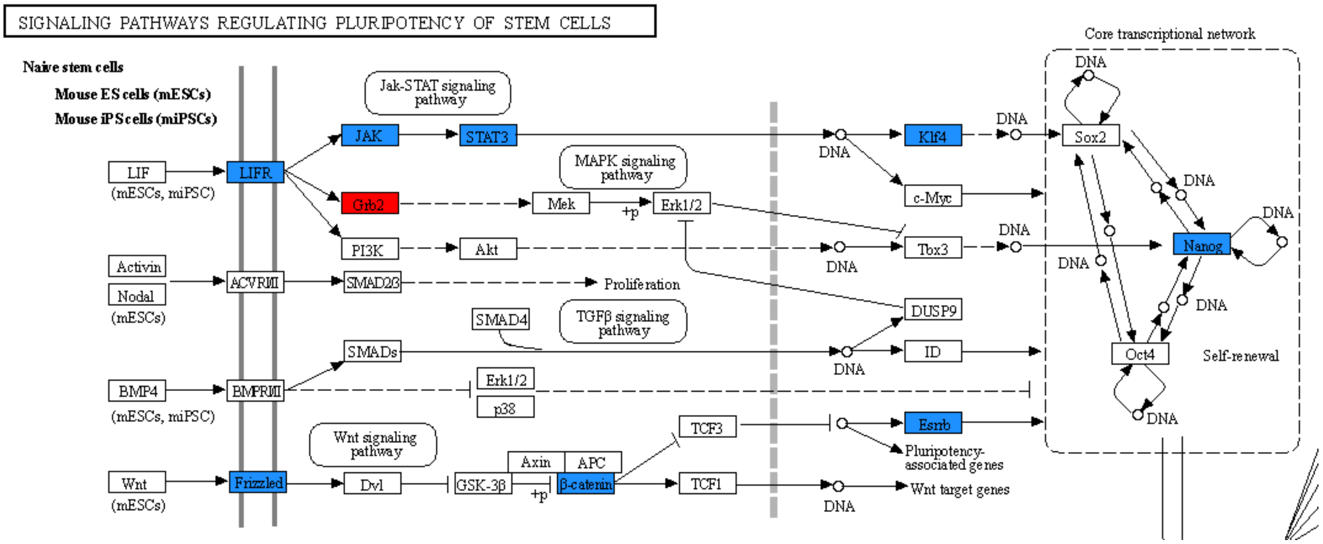
SIGNALING PATHWAYS REGULATING PLURIPOTENCY OF STEM CELLS
Naive stem cells
Mouse ES cells (mESCs)
Mouse iPS cells (miPSCs)
Core transcriptional network
Jak-STAT signaling pathway
MAPK signaling pathway
TGFβ signaling pathway
Wnt signaling pathway
LIF
(mESCs, miPSC)
LIFR
JAK
STAT3
Grb2
Mek
+p
Erk1/2
PI3K
Akt
Activin
Nodal
(mESCs)
ACVRI/II
SMAD2/3
Proliferation
SMAD4
SMADs
BMP4
(mESCs, miPSC)
BMPRI/II
Erk1/2
p38
Wnt
(mESCs)
Frizzled
Dvl
Axin
APC
GSK-3β
+p
β-catenin
TCF3
TCF1
DNA
Klf4
c-Myc
Tbx3
DUSP9
ID
Esrrb
Pluripotency-associated genes
Wnt target genes
Sox2
Nanog
Oct4
Self-renewal

F

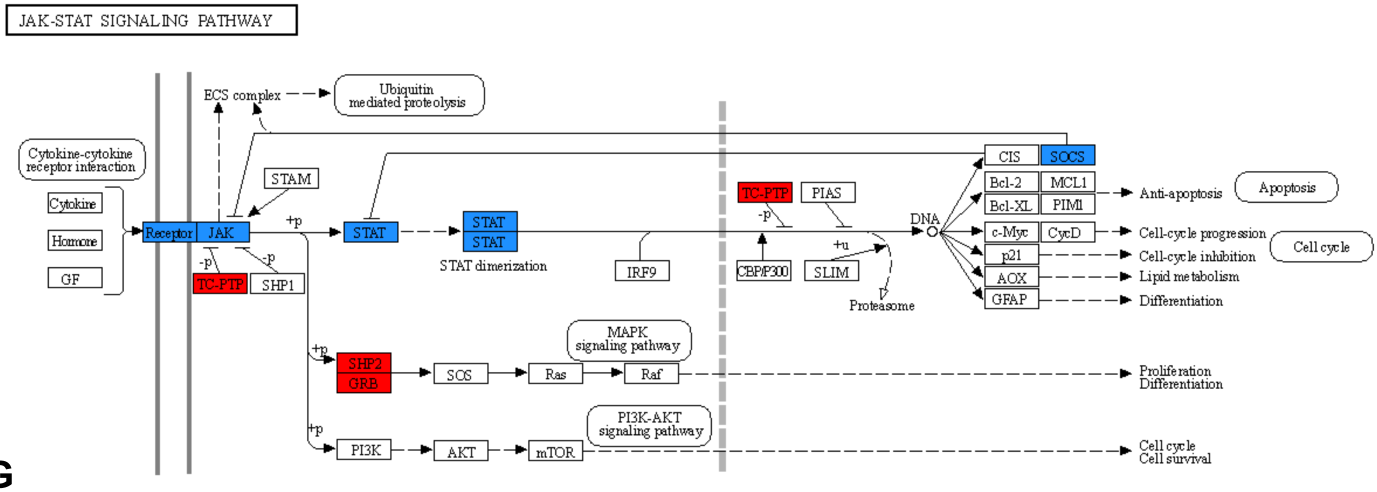
JAK-STAT SIGNALING PATHWAY
ECS complex
Ubiquitin mediated proteolysis
Cytokine-cytokine receptor interaction
Cytokine
Hormone
GF
Receptor
JAK
STAM
+p
-p
TC-PTP
SHP1
STAT
STAT
STAT
STAT dimerization
IRF9
TC-PTP
PIAS
CBP/P300
SLIM
+u
Proteasome
DNA
CIS
SOCS
Bcl-2
MCL1
Bcl-XL
PIM1
c-Myc
CycD
p21
AOX
GFAP
Anti-apoptosis
Apoptosis
Cell-cycle progression
Cell-cycle inhibition
Cell cycle
Lipid metabolism
Differentiation
SHP2
GRB
SOS
Ras
Raf
MAPK signaling pathway
Proliferation
Differentiation
PI3K
AKT
mTOR
PI3K-AKT signaling pathway
Cell cycle
Cell survival

G

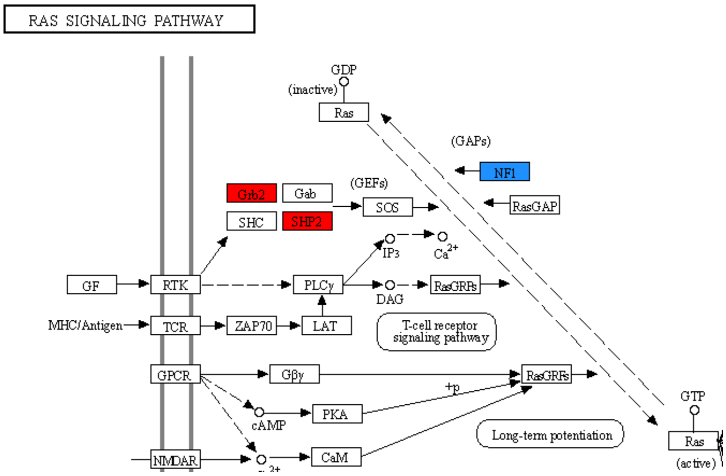
RAS SIGNALING PATHWAY
GDP
(inactive)
Ras
(GAPs)
NF1
RasGAP
Grb2
Gab
(GEFs)
SOS
SHC
SHP2
IP3
Ca2+
GF
RTK
PLCγ
DAG
RasGRPs
MHC/Antigen
TCR
ZAP70
LAT
T-cell receptor signaling pathway
GPCR
Gβγ
RasGRFs
+p
cAMP
PKA
NMDAR
CaM
Long-term potentiation
GTP
Ras
(active)

Figure. S2

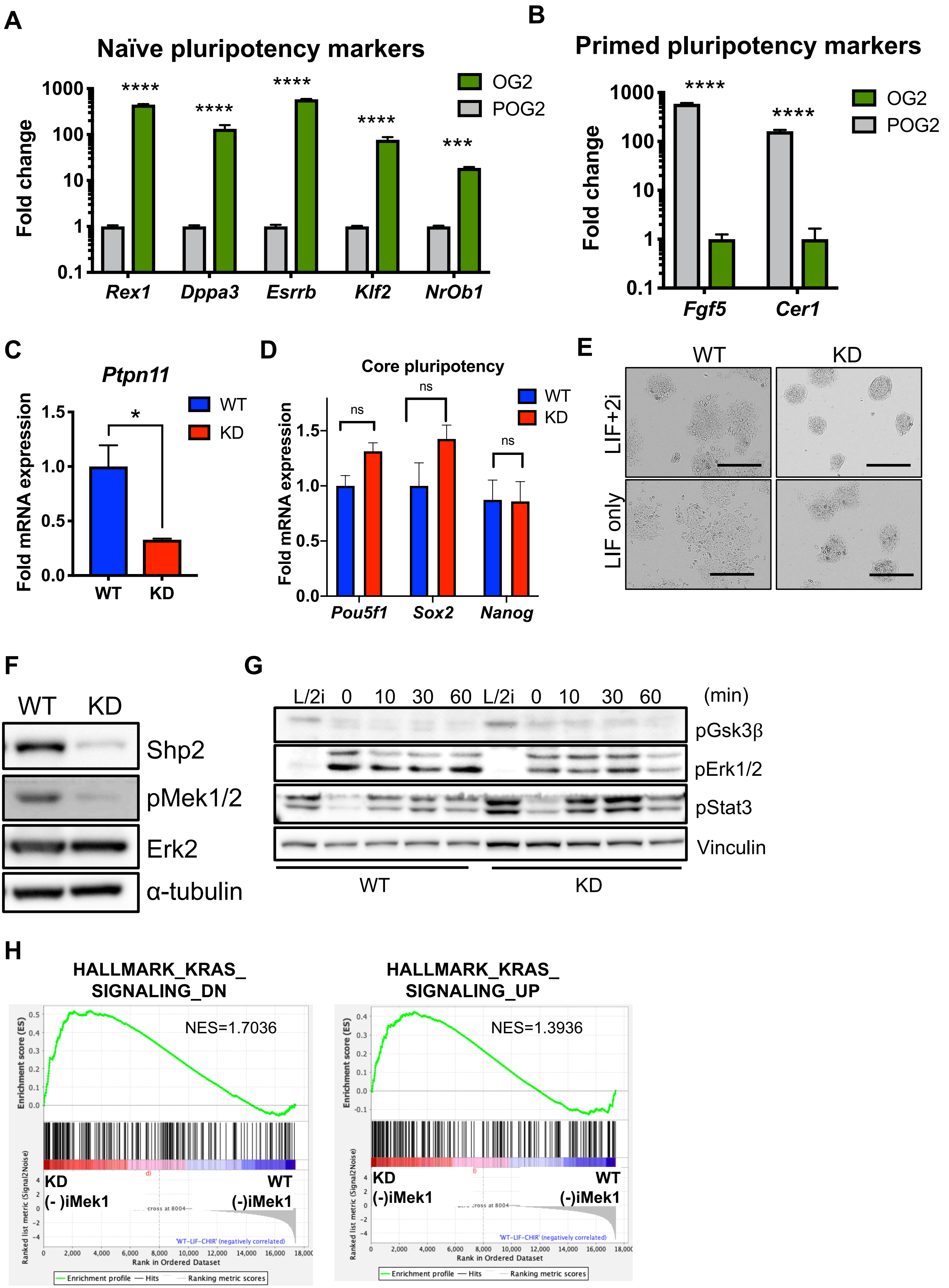
A
Naïve pluripotency markers
Fold change
OG2
POG2
****
****
****
****
***
Rex1
Dppa3
Esrrb
Klf2
NrOb1
B
Primed pluripotency markers
Fold change
OG2
POG2
****
****
Fgf5
Cer1
C
Ptpn11
Fold mRNA expression
WT
KD
*
D
Core pluripotency
Fold mRNA expression
WT
KD
ns
ns
ns
Pou5f1
Sox2
Nanog
E
WT
KD
LIF+2i
LIF only
F
WT
KD
Shp2
pMek1/2
Erk2
α-tubulin
G
L/2i 0 10 30 60 L/2i 0 10 30 60 (min)
pGsk3β
pErk1/2
pStat3
Vinculin
WT
KD
H
HALLMARK_KRAS_SIGNALING_DN
NES=1.7036
KD (-)iMek1
WT (-)iMek1
HALLMARK_KRAS_SIGNALING_UP
NES=1.3936
KD (-)iMek1
WT (-)iMek1

Figure S3

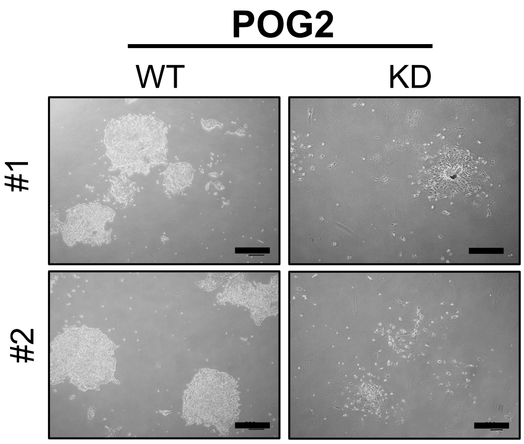

Figure S4

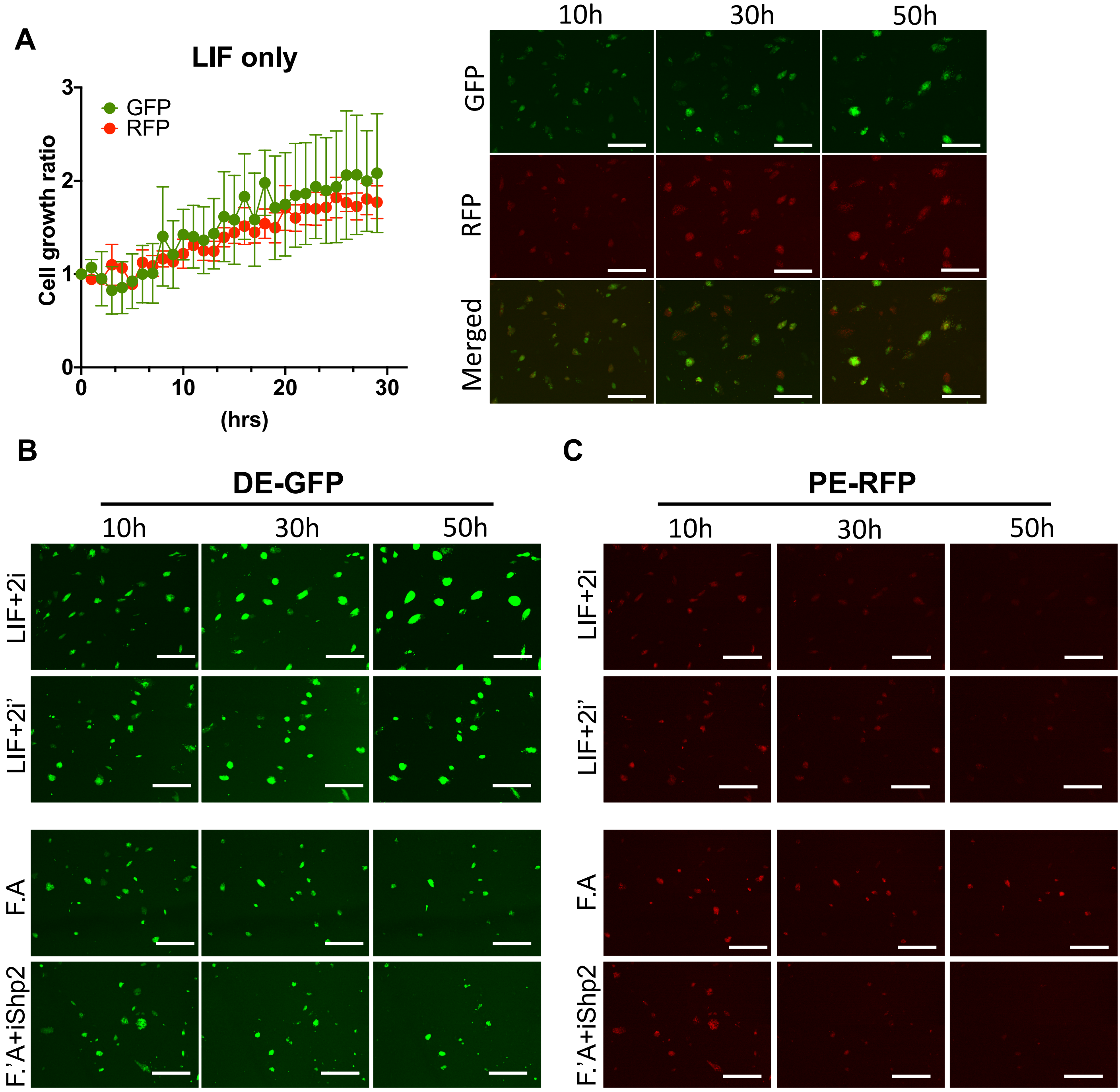
A
LIF only
GFP
RFP
Cell growth ratio
(hrs)
0
1
2
3
0
10
20
30
10h
30h
50h
GFP
RFP
Merged
B
DE-GFP
10h
30h
50h
LIF+2i
LIF+2i'
F.A
F.'A+iShp2
C
PE-RFP
10h
30h
50h
LIF+2i
LIF+2i'
F.A
F.'A+iShp2
